# CAR-T Therapy in GBM: Current Challenges and Avenues for Improvement

**DOI:** 10.3390/cancers15041249

**Published:** 2023-02-16

**Authors:** Ayush Pant, Michael Lim

**Affiliations:** 1 Department of Neurosurgery, School of Medicine, Johns Hopkins University, Baltimore, MD 21218, USA; 2 Department of Neurosurgery, School of Medicine, Stanford University, Stanford, CA 94305, USA

**Keywords:** CAR-T cells, immunotherapy, glioblastoma, exhaustion, immunosuppression

## Abstract

**Simple Summary:**

Clinical trials in glioblastoma (GBM) using CAR-T cells have not yielded tangible results. However, clinical and immunological observations from the trials have provided key insights into ways to limit toxicities and extend the antitumor efficacy of CAR-T cells. Incorporating recent notable strides in CAR-T engineering strategies could further boost CAR-T cytotoxicity against glioblastoma, increase persistence of CAR-T cells in vivo, and reduce off-target and on-target off-tumor effects. Here, we review the challenges highlighted by past clinical trials of CAR-T cells in GBM and review recently developed strategies that have the potential to overcome them.

**Abstract:**

Completed clinical trials of CAR-T cells in glioblastoma (GBM) have revealed key challenges that limit their efficacy. These include incomplete antigen coverage, downregulation of target antigen in response to therapy, exposure to immunosuppressive cells and cytokines in the tumor microenvironment and exhaustion of CAR-T cells. To overcome these challenges, CAR-T cells have been modified to maximize effector function and resist immunosuppression in the tumor while limiting toxicities to the host. Adoption of these novel CAR-T strategies in GBM can overcome the “cold tumor” phenotype of GBM and trigger an inflammatory cascade that maximizes tumor clearance and minimizes CAR-T dysfunction. To achieve this, understanding and harnessing the antigenic, metabolic and immunological composition of GBM is crucial. Here we review the findings from completed clinical trials of CAR-T cells in GBM as well as novel strategies that could improve CAR-T survival and function in the tumor.

## 1. Introduction

Glioblastoma (GBM) is the most aggressive primary brain tumor with a median overall survival of only 12 to 15 months despite aggressive treatment with surgical resection, chemotherapy, and radiation therapy [1]. While the use of immune checkpoint inhibitors (ICIs) has greatly improved the treatment of tumors, such as urological cancers, melanoma and non-small cell lung cancer, trials of ICIs, such as PD-1 blockade, have been disappointing in GBM [2,3]. While combinations of multiple checkpoint inhibitors are being tested in ongoing clinical trials, significant barriers remain that necessitate innovative solutions.

**Figure 1:**
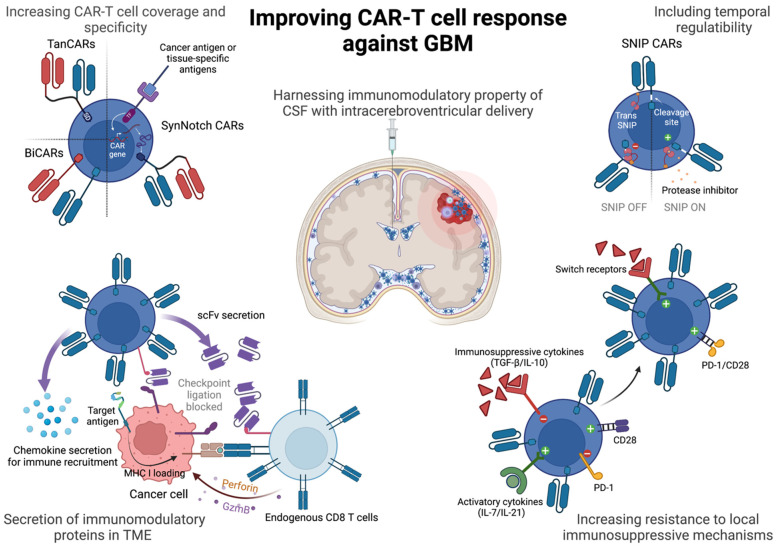
Strategies to improve CAR-T cells against GBM. CAR-T cells can be improved by adding greater antigen coverage against GBM, engineering tunability of response, resistance to exhaustion and immunosuppressive cytokines in the GBM tumor microenvironment. Intracerebroventricular route of administration of CAR-T cells might further licence CAR-T cells for long-term persistence and memory phenotype. SD = signaling domain comprising CD3ζ and co-stimulatory domains of CD28, ICOS, 4-1BB or OX-40. TF = transcription factor. scFv = single chain variable fragment. GzmB = granzyme B. TGF-β = transforming growth factor-β. PD-1 = programmed death-1. Created with BioRender.com.

One of the strategies that has been weaponized against malignancies include the use of chimeric antigen receptor T cells, or CAR-T cells, that have been genetically engineered to express chimeric receptors to recognize a target cell antigen. CARs have a target-binding extracellular domain derived from variable regions of antibodies (single-chain variable fragment or scFv), a flexible hinge and transmembrane spacer to anchor the CARs to the membrane and an intracellular domain derived from signaling domains of CD3ζ and additional co-stimulatory proteins derived from OX-40, 4-1BB, CD28 or ICOS either alone or in combination, to relay an activation signal upon antigen recognition [4]. Unlike conventional CD8 T cells that rely on presentation of processed peptides on the MHC-I molecule of antigen-presenting cells or cancer cells, CAR-T cells can bind to antigens directly on the target cell and relay a potent activation signal to trigger cytotoxicity against the antigen-bearing cell. This strategy has allowed for the generation of tumor-specific CAR-T cells (with added genetic modifications), multifold expansion prior to infusion in patients and greater amplification of the activation cascade upon antigen encounter. So far, hematological malignancies, such as leukemias, have shown impressive clinical response with the use of CD19 CAR-T cells [5,6], but successfully using CAR-T cell therapy for solid tumors has been rife with challenges. In this review we will discuss said challenges in treating GBM with CAR-T cells and highlight recent strides in CAR-T engineering that have the potential to significantly improve anti-GBM therapy (Figure 1).

## 2. Completed Clinical Trials of CAR-T Cells in GBM Highlight Key Challenges

GBM is characterized by extensive infiltration of immunosuppressive immune populations [7,8], aggressive tumor invasion [9], intratumoral and intertumoral heterogeneity of cellular states and driver mutations and antigens [10,11]. Neftel et al. have shown that malignant cells in GBM can be found in one of four states—neural progenitor-like, oligodendrocyte progenitor-like, astrocyte-like and mesenchymal-like states [12]. These cellular programs were not fixed either but displayed plasticity among them. While genetic drivers could determine these states, interaction with immune subsets, such as myeloid cells could also influence cells states, such as by promoting mesenchymal-like cell state [13]. Hematological malignancies, such as B cell acute lymphoblastic leukemia where the antigen CD19 is ubiquitously expressed on B cells, pose few challenges to CAR-T cells. Despite this, acquired mutations and alternative splicing of the tumor antigen can evade CAR recognition, as has been reported in a subset of patients with B-cell neoplasms [14]. GBMs show a greater target antigen heterogeneity that would make antigen coverage with CAR-T cells more challenging and clonal expansion of antigen-negative cancer cells or downregulation of the target antigen could thwart CAR-T therapy even in cases of transient antitumor response. Unfavorable conditions, such as an abundance of immunosuppressive myeloid cells and cytokines, lack of available glucose and upregulation of checkpoints, further limit CAR-T efficacy. Completed clinical trials with CAR-T cells targeting IL13Rα2, EGFRvIII and HER2 highlight some of these key challenges, while offering potential solutions to overcome them with the incorporation of innovative strategies in CAR-T engineering (Table 1).

### 2.1. EGFRvIII

Epidermal growth factor receptor (EGFR) is highly expressed in different tumor-types but presents a specificity challenge, as it is also found in normal tissues. However, the presence of epidermal growth factor vIII, a deletion mutant form of EGFR, on cancer cells in glioma and breast cancers presents a unique opportunity to target these cells specifically without cross-reactivity. The EGFRvIII mutant is generated during rearrangement of the EGFR gene, deleting an in-frame 801 base pair segment comprising exons 2–7 and making the receptor constitutively active [15]. Analysis of GBM biopsy revealed EGFRvIII is found in more than 50% of GBM [16] making them a potent target for tumor regression using CAR-T cells.

In a first-in-human clinical trial, O’Rourke et al. used a single dose of EGFRvIII-CAR-T cells injected systemically in 10 patients with EGFRvIII-positive recurrent GBM [17]. Although clinical benefit was not observed in this study, surgical intervention of seven of the patients allowed comparison of the tumor microenvironment (TME) after CAR-T therapy. CAR-T cells were found to infiltrate the tumor from systemic circulation, but there was also increased infiltration of regulatory T cells (Tregs) into the tumor and increased expression of inhibitory molecules, such as IDO (secreted by immunosuppressive myeloid cells), IL-10, TGFb and PD-L1, in the tumor in some patients. Loss of the EGFRvIII antigen was also observed in the patients, which along with the increase in immunosuppressive milieu of the tumor, likely resulted in attenuation of CAR-T cell efficacy. In another study, Goff et al. used preparative chemotherapy, which can reduce the burden of myeloid derived suppressor cells (MDSCs) and Tregs, before adoptive transfer of CAR-T cells to 18 patients and used IL-2 infusions to support CAR-T expansion [18]. Despite the greater persistence of CAR-T cells in this study, most likely due to the depletion of MDSCs and Tregs, there were no objective responses. These results show that making CAR-T cells resistant to the unique immunosuppressive background of GBM and increasing antigen coverage would tremendously improve their functionality in GBM.

### 2.2. IL13Rα2

IL13Rα2 is a high affinity IL13 receptor that is overexpressed by more than half of GBM cases and associated with poor patient survival [19]. It is not expressed on normal brain tissue making them an ideal target for CAR-T cells. A study by Brown et al. explored the use of CAR-T cells targeting IL13Rα2 as a pilot first-in-human study in recurrent GBM [20]. Despite transient antitumor activity in patients, including a case of dramatic clinical response for 7.5 months since first CAR-T treatment, patients eventually succumbed to the tumors, most likely due to observed downregulation of the target antigen IL13Rα2 and dominance of antigen-negative cells [21].

Recent studies using modified IL13Rα2-CAR-T cells have illuminated novel strategies to incorporate drug resistance and in vivo trackability in CAR-T cells in glioma. To reduce the time needed to generate autologous cell-based CAR-T therapy, Brown et al. generated off-the-shelf CAR-T cells that could target IL13Rα2 in GBM patients [22]. Dexamethasone, an immunosuppressive glucocorticoid, was administered to mitigate cerebral edema observed in GBM patients and to protect against the rejection of allogenic CAR-T cells. To reduce the detrimental impact of dexamethasone on persistence of CAR-T cells in the tumor, the cells were modified with biallelic inactivation of the glucocorticoid receptor gene with zinc finger nuclease prior to infusion. Although these modified CAR-T cells did not result in an objective clinical response, they displayed dexamethasone-resistant effector activity, suggesting that the vulnerability of T cells to the lymphopenic effects of chemotherapy could be overcome with additional modifications of CAR-T cells to make them resistant, such as the incorporation of efflux pumps. These GR-modified IL13Rα2 CAR-T cells have been further modified to express herpes simplex virus type-1 thymidine kinase (HSV1-tk) to allow the positron emission tomography (PET) imaging of CAR-T cells through phosphorylation and intracellular trapping of the herpes drug penciclovir that is radiolabeled with fluorine-18 (9-[4-[18F]fluoro-3-(hydroxymethyl)butyl]guanine ([18F]FHBG)) [23]. Tracking the dynamics of CAR-T infiltration with PET can provide a sensitive readout to evaluate strategies aimed at improving CAR persistence in vivo.

### 2.3. HER2

A member of the human epidermal growth factor receptor family, HER2, does not bind to a ligand for activation but instead forms heterodimers with ligand-activated EGFR or homodimers with other HER2 molecules on the cell surface making amplifications of HER2 expression potentially tumorigenic [24]. In vitro experiments have shown HER2 CAR-T cells can successfully kill glioma cells [25], but more in depth in vivo studies need to be conducted as HER2 is expressed on normal tissues as well [26]. Ahmed et al. engineered virus-specific T cells to express CARs to further extend persistence of CAR-T cells in a phase one clinical trial of HER2 CAR-T cells in GBM patients [27]. Due to the presence of latent viral antigens from cytomegalovirus (CMV), Epstein–Barr virus (EBV) or adenovirus (Adv) in the body, using virus-specific T cells to express CARs can allow robust co-stimulation by APCs presenting latent viruses, leading to greater CAR-T persistence and activity in vivo. These CAR-VSTs (virus-specific T cells) persisted in the peripheral blood of patients up to one year after infusion, although they did not expand most likely due to the absence of lymphodepleting chemotherapy prior to infusion. A total of 8 patients out of 17 had clinical benefits with partial response or stable diseases.

## 3. Strategies to Improve CAR-T Response

### 3.1. Bispecific CAR-T Cells

A strategy to increase the target coverage of CAR-T cells is the simultaneous use of multiple CAR specificities to avoid tumor escape. Given the heterogeneity of GBMs, Hegde et al. used mathematical modeling to capture the antigen expression heterogeneity of GBM in order to predict the optimum combination of antigen-specificities that would yield the greatest therapeutic benefit in patients [28]. They found that the odds of capturing the bulk of GBM tumor cells with the co-targeting of two antigens was far better than with targeting a single entity and similar to targeting three antigens. Bispecific T cells expressing both HER2 and IL13Rα2 CARs exhibited greater activation and antitumor activity in in vitro and ex vivo experiments and in an orthotopic xenograft model of human GBM compared to HER2 CAR-T cells alone, IL13Rα2 CAR-T cells alone or pooled HER2 and IL13Rα2 CAR-T cells.

Recently, Tian et al. highlighted a novel approach incorporating CITE-seq (Cellular Indexing of Transcriptomes and Epitopes by Sequencing) to identify high-activity CARs before combining them into bicistronic CAR-T cells [29]. To identify optimum CARs against two antigens in neuroblastoma, GPC2 and CD276, that together are expressed in 95% of neuroblastoma samples, they designed a competition assay pooling eight clones of anti-GPC2 CAR-T cells and six anti-CD276 CAR-T cells and cultured them with and without target tumor cells for 24 h. Prior to single cell sequencing, the CAR-T cells were incubated with an antibody-derived tag (for simultaneous protein expression analysis of activation and differentiation markers, to corroborate sequencing results). This allowed identification of CARs that promote maximum proliferation and activation upon target cell exposure. In addition to preventing antigen escape, bicistronic CAR-T cells thus engineered with a P-COCC approach (pooled competitive optimization of CAR by Cite-seq) also exhibit greater persistence and less exhaustion in animal models compared to unispecific CAR-T cells. With the transient antiglioma response observed in the clinic with the above discussed single-antigen targeting CAR-T cells, there is a rationale to combine novel engineering strategies with newer sequencing tools to create and identify high affinity clones of CARs that can be used together against GBM to overcome antigen heterogeneity. Recently, Hegde et al. developed tandem CAR-T cells (tanCAR-T) that express a CAR that joins the HER2 binding scFV with the IL13Rα2-binding IL13 mutant protein for simultaneous engagement of HER2 and IL13Rα2 with the aim of increasing the overall avidity of interaction [30]. Upon encounter of both antigens on tumor cells, there was superadditive T cell activation in TanCAR-T cells compared to biCAR-T cells and longer lasting cytotoxicity. TanCAR-T cells also greatly outperformed biCAR T cells in in vivo antitumor efficacy.

### 3.2. Engineering Temporal and Spatial Control over CAR-T Cell Activity

Severe CNS toxicity following CAR-T therapy has been noted in patients during treatment of refractory B-cell malignancy with CD19 CAR-T cell therapy [31]. Robust CAR-T cytotoxicity against tumor cells can trigger a systemic inflammatory response called cytokine release syndrome, which can cause lethal neurotoxicity in some patients. The blood–brain barrier (BBB) has been observed to be permeable in such patients, likely due to the vicious cycle of systemic cytokines penetrating the brain, activating brain vascular pericytes, causing secretion of endothelium-activating cytokines from pericytes and promoting further breakdown of the blood–brain barrier and leakiness [32]. While the major first-in-human clinical trials in GBM have not shown similar levels of toxicities [33], most likely due to limited efficacy of CAR-T cells, any improvement in CAR-T cell functionality against GBM will need to be countered with fail-safe mechanisms to prevent CAR-T-related encephalopathy syndrome (CRES).

Engineering regulatability into CAR-T cells can have multiple advantages. Ability to turn CAR-T cells off and on after infusion can allow temporal control over toxicity and effector function. Turning off CAR-T cell effector function with pharmaceutical intervention can be useful and lifesaving upon onset of toxicity. However, to avoid compromising antitumor efficacy, they will need to be turned on again once toxicities have subsided. Labanieh et al. [34] recently developed protease-regulated SNIP (signal neutralization by an inhibitable protease) CAR-T cells that will remain in an ON state in the presence of a protease inhibitor and can be turned OFF following drug withdrawal. The hepatitis C virus NS3 protease (NS3p) was incorporated as a membrane-bound cytoplasmic protein that was in trans (not on the same molecule) to the CAR, which contained a NS3p-cleavage site (CS) between the transmembrane and signaling domain. After administration of grazoprevir, an NS3p inhibitor, cleavage of the CAR is prevented, and CARs continue to signal upon antigen counter. Upon withdrawal of the drug, cleavage of the CS occurs, and the CAR-T state will be in an OFF state where it can no longer exhibit cytotoxicity and cytokine secretion.

SNIP CAR-T had greater cytotoxicity against tumors and in vivo persistence compared to constitutively active CAR-T cells and were more likely to differentiate into a memory phenotype. Most notably, SNIP CAR-T cells had diminished expression of markers associated with T cell exhaustion, most likely due to avoidance of chronic activation of T cells. SNIP CAR-T cells were also superior at targeting CNS tumors, as evidenced by their performance against medulloblastoma tumors in murine models.

On-target off-tumor cross reactivity of CAR-T cells can lead to lethal toxicities if CARs recognize normal nonpathogenic tissue expressing the target antigen [35]. In order to prevent on-target off-tumor toxicity and incomplete killing by CAR-T cells, Choe et al. engineered CAR-T cells to include SynNotch receptors that activate a transcriptional factor upon recognition of the “priming antigen”, which promotes the transcription of CAR genes that will recognize the target antigens locally [36,37]. Using this system, they created α-EGFRvIII synNotch–α-EphA2/IL13Rα2 CAR- T cells where EGFRvIII acts as the priming antigen to turn on expression of the tandem α-EphA2/IL13Rα2 CARs (similar to tanCARs mentioned above), confining CAR-T cell function spatially to EGFRvIII expressing tissues. These synNotch CAR-T cells can then kill the priming EGFRvIII+ cell if they express α-EphA2 or IL13Rα2 (cis killing) or kill neighboring EGFRvIII-cells that express α-EphA2 or IL13Rα2 (trans killing). This circuitry prevents activation of CAR-T cells in normal tissue where the priming antigen EGFRvIII is absent and prevents antigen escape as both EGFRvIII+ and EGFRvIII-GBM cells are likely to express at least one of the target antigens. Choe et al. also created tissue-specific α-MOG synNotch–α-EphA2/IL13Rα2 CAR-T cells that recognize the priming antigen myelin oligodendrocyte glycoprotein (MOG), which is found on the myelin sheath of neurons. These tissue-specific synNotch CAR-T cells displayed potent anti-α-EphA2/IL13Rα2 activity and were confined to MOG-expressing tissues, which acted as priming cells. Furthermore, compared to constitutive CAR-T cells, these synNotch CAR-T cells displayed reduced antigen-induced tonic signaling and thereby exhibited less exhaustion and longer persistence in vivo.

Incorporating mechanisms of temporal and spatial control in infused anti-GBM CAR-T cells could not only avoid off-target and on-target/off-tumor toxicities but also prevent chronic activation-induced exhaustion in the TME, leading to a greater persistence of CAR-T cells and an increased memory phenotype to prevent tumor recurrence.

### 3.3. Engineering Resistance to Immunosuppressive Cytokines in the TME

The GBM TME is a hostile environment to CTLs, marked by high infiltration of immunosuppressive myeloid cells [7], Tregs [8] and the presence of immunosuppressive cytokines [38], making it a “cold” tumor with minimum inflammatory properties [39]. Engineering resistance to the unique vulnerabilities found in GBM into CAR-T cells might be able to extend their persistence and effector function. In addition to CARs, other transgenes can be added to CAR-T cells to trigger a cascade of inflammatory changes in GBM to make it a “hot” tumor. Adachi et al. modified CAR-T cells to express IL-7, a cytokine important for T cell proliferation and survival, and CCL19, a chemokine for DC and T cell migration. Consistent with their role in maintaining T-cell zone in lymph nodes, expression of these cytokines from CAR-T cells promoted the infiltration of T cells and DCs into mastocytoma, boosting antitumor efficacy compared to conventional CAR-T cells [40]. Such an approach in GBM could make the TME “hot” by recruiting other arms of the myeloid and lymphoid immune system and triggering a cascade of CAR-T cell-mediated cancer cell death, epitope spreading, antigen uptake by intratumoral APCs and activation of endogenous T cells as well, which are indispensable to the success of CAR-T cells as evidenced by the dampening of the therapeutic effect of CAR-T cells when endogenous T cells of recipient tumor-bearing mice are ablated [40]. Due to the effect of IL-15 on CD8 T cell activation and the generation of memory response, Krenciute et al. engineered anti-GBM IL13Rα2.IL-15 (secreting) CAR-T cells, which they found to be superior in proliferative capacity, cytokine production and cytotoxicity [41].

CAR-T cells have also been engineered to target specific vulnerabilities of tumors. For the treatment of tumors that abundantly secrete IL-4, a Th2 polarizing cytokine that inhibits Th1 polarized effector T cell response needed for antitumor activity, Leen et al. created an “inverted receptor” where the extracellular domain of IL-4 was fused with the intracellular domain of IL-7 receptor so that IL-4 would trigger activation of IL-7 signaling cascade, promoting T cell activity and proliferation [42]. Similarly, Wang et al. fused IL-4 exodomain to IL-21 endodomain to simultaneously prevent immunosuppression by IL-4+ solid tumors and to promote IL-21 mediated enhancement of T cell cytotoxicity [43]. In order to target GBM-specific immunosuppressive pathways, creating inverted cytokine receptors with exodomain from the TGF-b receptor or IL-10R and ectodomain from IL-7 or IL-21 might combat the immune-suppressive role of these cytokines as these are abundantly secreted by GBM and play an important role in attenuating T cell effector function. [44].

In addition to making CAR-T transgenes, adjuvants can also be used to achieve concurrent activation of CAR-T cells in the tumor while decreasing the Treg- and MDSC-burden in the tumor, which heavily infiltrate GBMs. One such strategy is intratumoral delivery of IL-12 in a preclinical model of GBM, which increases CAR-T activation and increases the ratio of conventional CD4 T cells to Tregs and increases myeloid activation in the tumor [45]. Injection of oncolytic adenovirus with CXCL11 gene is yet another adjuvant that has been used along with anti-GBM CAR-T cells to improve efficacy and to decrease MDSC and Treg infiltration into the tumor in murine models [46].

### 3.4. Engineering Resistance to Exhaustion

Tumor infiltrating CD8 T cells and CAR-T cells express inhibitory checkpoints, such as PD-1 and CTLA-4, which can suppress T cell activity upon binding their ligand expressed on cancer cells and tumor-infiltrating myeloid cells [47]. Using ICIs have become a major pillar of immunotherapy with notable successes in many cancers, and checkpoint blockade has been observed to boost CAR-T function in preclinical models as well [47]. However, ICIs have not been effective in GBM, and the efficacy of combining ICIs with CAR-T cells remain to be seen. Modifying PD-1 in CAR-T cells is a potential strategy to preclude systemic administration of anti-PD-1 antibodies, which can have multiple toxicities and boost CAR-T function in the tumor. Cherkassry and Morello et al. engineered CAR-T cells to express PD-1 that lacked an intracellular signaling domain and noted that CAR-T cells with such dominant negative PD-1 (sequestering PD-L1 from binding intact PD-1) had better in vivo cytotoxicity against mesothelin+ tumors in mice compared to CAR-T cells with full-length PD-1 [47]. Liu et al. modified PD-1 in CAR-T cells by fusing the extracellular and transmembrane domain of PD-1 with the intracellular signaling domain of CD28 so that inhibitory signal in the TME could be switched to an activatory signal [48]. Such a “switch-receptor” CAR-T cell yielded better tumor control than the concomitant administration of anti-PD-1 with CAR-T cells. Due to the high expression of checkpoints, such as PD-1, LAG-3, TIM-3 and TIGIT as well as their ligands in GBM [49], converting ubiquitous inhibitory signals in the TME to stimulatory signal can greatly improve antiglioma cytotoxicity. CAR-T cells have also been engineered to secrete a single-chain variable fragment that blocks PD-1 (both in an autocrine and paracrine fashion) [50] or anti-PD-L1 [51]. This approach of limiting the secretion of antibodies against checkpoint/checkpoint ligands locally in the TME can also prevent immune-related adverse events (irAEs) associated with systemic administration of immunotherapy.

Another characteristic of the TME is the lack of glucose for immune cells due to the high level of aerobic glycolysis and lactate production in cancer cells, even in the presence of oxygen (the Warburg effect) [52]. MDSCs and macrophages infiltrate GBMs heavily [7] and can further limit glucose availability through increased uptake [53]. This can limit the cytotoxic function of CAR-T cells in the tumor. Ho et al. discovered that phosphoenolpyruvate (PEP), a glycolytic metabolite, sustains CD4 T cell effector function and is insufficiently produced during glucose deprivation and that overexpression of phosphoenolpyruvate carboxykinase 1 (PCK1, which converts oxaloacetate into PEP in gluconeogenesis) can bypass glucose deprivation and promote T cell function [54]. Adopting similar strategies in CAR-T cells against GBM can help prevent metabolic exhaustion, which precedes phenotypic exhaustion [55].

### 3.5. Utilizing the Unique Anatomical and Immunological Niche of GBMs for CAR-T Delivery

A phase I trial of GD2 CAR-T cells in children and young adults with midline glioma has demonstrated a previously unappreciated role of the route of CAR-T administration in shaping inflammatory milieu in the CNS. [56] Infusions of CAR-T cells intracerebroventricularly (i.c.v.) resulted in lesser systemic toxicity compared to i.v. infusions. The i.v. infusions were correlated with increased immunosuppressive myeloid cell populations in the CSF, whereas i.c.v. administration resulted in greater immune-activating signature in myeloid cells. The i.c.v. infusions of CD19-CAR-T cells against CNS lymphoma were also found to be superior to i.v. administration in murine models [57]. Interestingly, control of concurrent systemic lymphoma was also improved with the i.c.v. administration compared to the i.v. route due to the role of CSF in favoring enhanced memory phenotype and reduce differentiation and exhaustion signatures of CAR-T cells [57]. The i.c.v. infusions were also better than the i.v. delivery of CAR-T cells in controlling breast to brain metastatic lesion xenografts in mice [58] and either comparable to or marginally better than intratumoral (i.c.) delivery of CAR-T cells in controlling breast metastatic brain tumors [58] and contralateral/distal multifocal GBM in mice [59]. Since multiple clinical trials have shown that i.c.v. administration is safe in humans [21,59], trials with a greater number of patients are needed to compare the effects of i.v., i.c. and i.c.v. route of administration on CAR-T activation, myeloid-mediated immunosuppression, persistence and memory function.

Furthermore, CAR-T therapy can be combined with novel strategies to enhance CAR-T cell homing to GBM. Ma et al. have targeted the vascular microenvironment of GBMs to increase CAR-T cell infiltration [60]. They identified p21-activated kinase 4 (PAK-4) as a regulator of aberrant vascularization in GBM and used pharmacological inhibitor of PAK-4 to normalize tumor vasculature. This lead to greater permeability of endothelial cells and increased CAR-T effector function in murine models of GBM [60]. This strategy can be leveraged to increase CAR-T retention and efficacy in the tumor following the i.v. administration of CAR-T cells.

## 4. Conclusions

CAR-T therapy has failed to show efficacy in GBM due to the same challenges that have thwarted immune checkpoint blockade. Tumor intrinsic factors, such as extensive heterogeneity in protein expression and mutational status, competition for limited nutrients, infiltration of CD8-supressing immune populations, such as Tregs and tumor-associated macrophages and secretion of immunosuppressive factors, can limit efficacy of endogenous T cells following immunotherapy as well as that of adoptively transferred CAR-T cells. Here, we discussed key factors behind the failure of CAR-T cells in clinical trials in GBM. Results from recent preclinical studies and clinical trials suggest that successful treatment of solid tumors, such as GBM, with CAR-T cells will rely on the intersection of multiomic approaches to comprehensively understand the TME (including the genetic, epigenetic, proteomic and metabolic facets) and astute genetic engineering to tap the unique vulnerability in each tumor type or patient. As an example, reciprocal CRISPR screening of CAR-T cells and GBM stem cells from co-cultures have revealed dependencies for effector function in CAR-T cells as well as resistance mechanisms among cancer cells [61]. Engineering modifications into CAR-T cells to negate resistance mechanisms based on these multiomics findings can yield tremendous clinical benefits to patients. Strategic incorporation of small molecules and adjuvants along with CAR-T therapy also has potential to greatly improve efficacy. As we discussed here, PAK-4 inhibitors can be used to improve the CAR-T infiltration of GBM [60]. Other small molecules, such as antagonists of inhibitor of apoptosis protein (IAP), have been recently shown to promote bystander killing of antigen-negative GBM cells by CAR-T cells through sensitization to CAR-T-derived cytokines [62], opening new avenues for overcoming tumor antigen heterogeneity. Furthermore, clinical trials have largely been conducted in patients with refractory or recurrent GBM who have failed standard of care therapy. Future clinical trials will be necessary to examine the efficacy of co-administering CAR-T cells during the standard of care therapy window or during neo/adjuvant immunotherapy.

## Figures and Tables

**Table 1:** Summary of clinical trials evaluating CAR-T cells in GBM.

Target Antigen	Trial	Phase	No. of Patients	Current Status	Notes
EGFRvIII	NCT02209376	I	10	Complete	Increased expression of inhibitory molecules and loss of EGFRvIII expression following therapy.
EGFRvIII	NCT01454596	I/II	18	Complete	Preparative chemotherapy used before adoptive transfer of CAR-T cells. IL-2 infusion used to support CAR-T expansion.
IL-13Rα2	NCT00730613	I	3	Complete	Transient antitumor activity observed
IL-13Rα2	NCT01082926	I	6	Complete	Suitable for allogeneic off-the -shelf use. Glucocorticoid receptor inactivated in CAR-T cells. Modified to allow PET imaging.
IL-13Rα2	NCT04003649	I	60	Recruiting	Administering with and without nivolumab and ipilimumab.
IL-13Rα2	NCT02208362	I	82	Active, not recruiting	Using memory enriched T cells for CAR-expression.
IL-13Rα2	NCT04661384	I	30	Recruiting	Treating leptomeningeal diseases from GBM, ependymoma or medulloblastoma.
HER2	NCT01109095	I	17	Complete	Virus-specific T cells engineered to express CAR-T to increase persistence.
HER2	NCT03389230	I	42	Recruiting	Using memory enriched CAR-T cells in grade II-IV glioma patients.
MMP2	NCT04214392	I	36	Recruiting	Using chlorotoxin expressing CAR-T cells to recognize GBM cells expressing putative receptor metalloproteinase 2 (MMP2).
B7-H3	NCT05366179	I	36	Recruiting	Targeting B7-H3 expression in GBM.
B7-H3	NCT05474378	I	39	Recruiting	Targeting recurrent IDH-wild type GBM patients.
